# Aqueous electrolyte-gated solution-processed metal oxide transistors for direct cellular interfaces

**DOI:** 10.1063/5.0138861

**Published:** 2023-04-10

**Authors:** Dong-Hee Kang, Jun-Gyu Choi, Won-June Lee, Dongmi Heo, Sungrok Wang, Sungjun Park, Myung-Han Yoon

**Affiliations:** 1 School of Materials Science and Engineering, Gwangju Institute of Science and Technology, Gwangju 61005, Republic of Korea; 2 Electrical and Computer Engineering, Ajou University, Suwon 16499, Republic of Korea

## Abstract

Biocompatible field-effect-transistor-based biosensors have drawn attention for the development of next-generation human-friendly electronics. High-performance electronic devices must achieve low-voltage operation, long-term operational stability, and biocompatibility. Herein, we propose an electrolyte-gated thin-film transistor made of large-area solution-processed indium–gallium–zinc oxide (IGZO) semiconductors capable of directly interacting with live cells at physiological conditions. The fabricated transistors exhibit good electrical performance operating under sub-0.5 V conditions with high on-/off-current ratios (>10^7^) and transconductance (>1.0 mS) over an extended operational lifetime. Furthermore, we verified the biocompatibility of the IGZO surface to various types of mammalian cells in terms of cell viability, proliferation, morphology, and drug responsiveness. Finally, the prolonged stable operation of electrolyte-gated transistor devices directly integrated with live cells provides the proof-of-concept for solution-processed metal oxide material-based direct cellular interfaces.

## INTRODUCTION

With the increasing demand for human-friendly bioelectronic devices, substantial effort has been devoted to exploring electronic materials for extracting/stimulating electrical signals from/to cells, aiming to understand the fundamentals of self-electroactive systems. Direct cellular interfaces are of particular interest in revealing the mechanism of electron–ion interactions, thus fostering the research and development of novel device platforms. Conventional multielectrode-array techniques^1–10^ have been widely used to measure cellular signals by capacitive coupling between metal electrodes and biological cells. Field-effect transistors based on three-terminal electrodes have also been introduced.^11–22^ Such developments engender more interest in the study of human–machine interfaces by leveraging low parasitic capacitance^11–22^ and self-amplifying functionalities to achieve high signal-to-noise ratios.^14,15,18–20^ In addition, accurate signal acquisition at the electroactive cellular interfaces of electronic devices can be achieved from the following characteristics: (1) low-voltage operation in aqueous solution to prevent water electrolysis, (2) long-term operational robustness, (3) biocompatibility at the cell–semiconductor interface, (4) cost-effective and large-area processibility, and (5) reusability. To date, organic electronics based on conjugated polymers, including polypyrrole,^23,24^ polyaniline,^25,26^ polythiophene,^27,28^ and poly(3,4-ethylenedioxythiophene):polystyrene sulfonate,^29–31^ allow the detection and/or stimulation of cellular activities via redox (i.e., de-/doping) reactions. Particularly, despite substantial advantages of organic mixed ionic–electronic conductors and corresponding electrochemical transistors, relatively low electrical characteristics, mechanical fragility, and possible swelling in aqueous media should be thoroughly addressed to obtain their long-term operation for stable cellular interfaces.

As a potential candidate for effective bioelectronic interfaces, solution-processed metal oxide-based semiconducting materials have drawn much attention^32–35^ owing to their high mobility^36–40^ and optical transparency,^41,42^ which are conducive to outperforming conventional amorphous silicon semiconductors. Moreover, their mass-productive solution processability and good environmental/operational stability enable efficient low-cost fabrication of high-quality electronic devices.^32–35^ In particular, metal oxide-based electrolyte-gated thin-film transistors (EGTFTs) containing aqueous electrolyte dielectrics as a gate-insulating medium allow long-term stable device operation at a very low bias because of the large areal capacitance (∼10 *μ*F/cm^2^) induced by electrical double-layer formation at the interface between electrolytes and active layer, which is 100-fold larger than those of typical solid-state dielectrics (∼0.005–0.5 *μ*F/cm^2^).^43–51^ Previously, we reported indium–gallium–zinc oxide (IGZO)-based EGTFTs operating under sub-0.5 V conditions in various aqueous solutions containing common ionic salts.^50^ To the best of our knowledge, IGZO-EGTFTs have not been tested for operation under physiological conditions, and their cytotoxicity to mammalian cells cultured on top has not been assessed.

Nonetheless, it remains challenging to obtain direct cellular interfaces that simultaneously secure long-term device operation and support cellular activity. To develop such bioelectronic interfaces, the following aspects should be comprehensively considered: (1) the cell-interfacing active materials should be optically transparent and mechanically stable/robust in typical culture media for electrical and optical measurement simultaneously; (2) the biological signals obtained at the interface with active channel should have sufficient amplitude to deliver the meaningful information; (3) the scalable fabrication of large-area device arrays and, possibly, their repeated use after reasonable cleaning steps should be developed;^13,17^ (4) the reliable cell viability and attachment on the active channel layer should be examined;^15,18,21^ and (5) the undisturbed cellular behaviors during the device operation need to be guaranteed with minimal protection/passivation layer inclusion.^11,12,14,18,19^

In this research, we developed a direct cellular interface based on solution-processed transparent IGZO-EGTFTs, which operates under sub-0.5 V conditions in aqueous cell culture media. We mainly focused on the proof-of-concept for sol-gel IGZO-based direct cellular bioelectronic interfaces where various types of cells (e.g., fibroblast, neurons) are directly cultured on the bare sol-gel IGZO surfaces or the as-fabricated EGTFTs without any chemical modification introduced. The large-area IGZO-EGTFT arrays were electrically characterized, in particular, focusing on transconductance, subthreshold swing, on-/off-current ratios, operational stability, etc. Furthermore, the potential application of IGZO-EGTFTs for various types of cellular interfaces was evaluated in terms of cell viability, proliferation, and growth analysis. Finally, the feasibility of IGZO-EGTFTs for bioelectronic interfaces and biosensors was carefully examined.

## RESULTS AND DISCUSSION

### Design and general performance of IGZO-EGTFTs

**FIG. 1.**
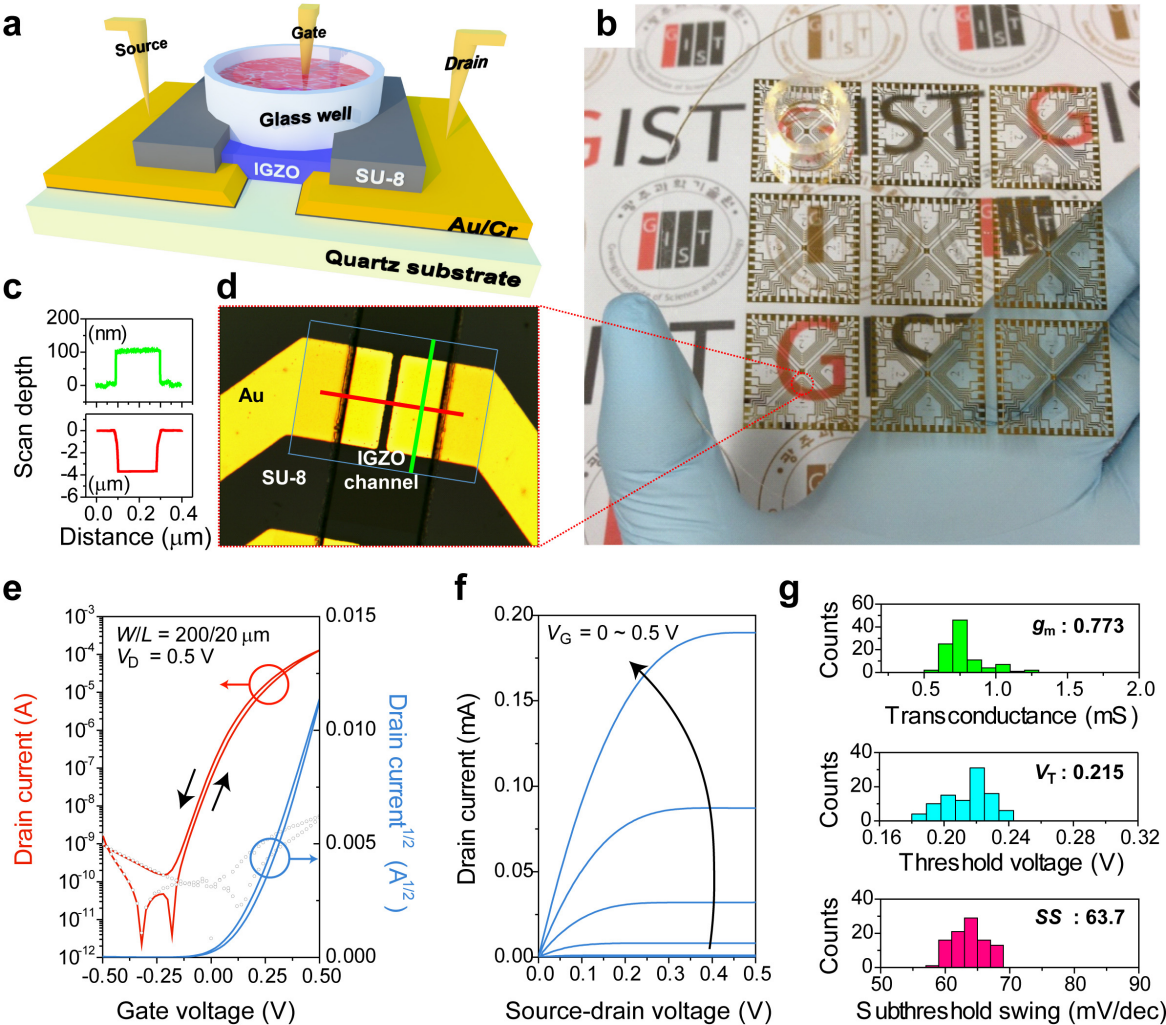
Fabrication and electrical characteristics of IGZO-EGTFTs. (a) Schematic of biocompatible IGZO-EGTFT devices. (b) Photograph of IGZO-EGTFT arrays on the 4-in. quartz wafer. (c) Thickness measurement of source and drain electrodes (green) and SU-8 passivation layer (red). (d) Optical microscopy image of channel region in an IGZO-EGTFT. The rectangle indicates areas of IGZO semiconductors, and the green and red lines indicate the traces of surface profilers. Representative (e) transfer (red; logarithmic, blue; square root drain currents) and (f) output curves (*V_G_* = 0–0.5 V with 0.1 V interval) of IGZO-EGTFTs with DMEM as gate electrolytes [200 and 20 *μ*m width (*W*) and length (*L*), respectively]. (g) Statistical distributions of maximum transconductance (*g*_m_), threshold voltages (*V*_T_), and subthreshold swings (*SS*). The error bars indicate the standard deviations out of 90 device measurements.

Figure 1(a) depicts a schematic illustration of the IGZO-EGTFT, while Fig. 1(b) shows a photograph of an array of 216 IGZO TFTs on a quartz substrate. Note that, to minimize the leakage-current path during operation, 100 nm-thick Au/Cr electrodes were deposited as bottom-contact source–drain electrodes under IGZO semiconductors in a staggered structure, and 4 *μ*m thick SU-8 passivation was defined to exposure-active areas of the semiconducting layer for electrical isolation [Figs. 1(c) and 1(d)]. To investigate the device operation under physiological conditions, we used cell culture media based on Dulbecco's modified eagle medium (DMEM) as electrolytes. DMEM is a widely used mammalian cell-culture medium and is composed of nutritional elements, including amino acids, inorganic salts, vitamins, and glucose in water as well as 10% fetal bovine serum (FBS) and antibiotics to maintain pH with osmotic balance, support the neutralization and growth of biological cells, and prevent fungal or bacterial contamination. Figures 1(e) and 1(f) show the representative transfer and output curves of IGZO-EGTFTs with the channel dimension of 200 and 20 *μ*m width (*W*) and length (*L*), respectively, operated at constant source–drain voltage (*V*_D_ = +0.5 V) in contact with DMEM. The EGTFTs showed impressive n-type electrical characteristics within a 0.5 V low-operation-voltage window and included high on-/off-current ratios of 10^7^, a transconductance (*g*_m_ *=* Δ*I*_D_/Δ*V*_G_; drain current is denoted as *I*_D_, and gate voltage is denoted as *V*_G_) as high as 0.773 ± 0.082 mS, threshold voltage (*V*_T_) of 0.215 ± 0.014 V, and subthreshold swing (*SS*) of 63.7 ± 2.65 mV/dec on average across over 90 devices with narrow distributions [Fig. 1(g)]. We also confirmed negligible changes in electrical characteristics caused by the extra additives (10% FBS and 1% penicillin/streptomycin), indicating that small amounts of FBS and antibiotics were ineffective and that no physical adsorption occurred at the IGZO surface (Fig. S1).

### Long-term operational stability of IGZO-EGTFTs

**FIG. 2.**
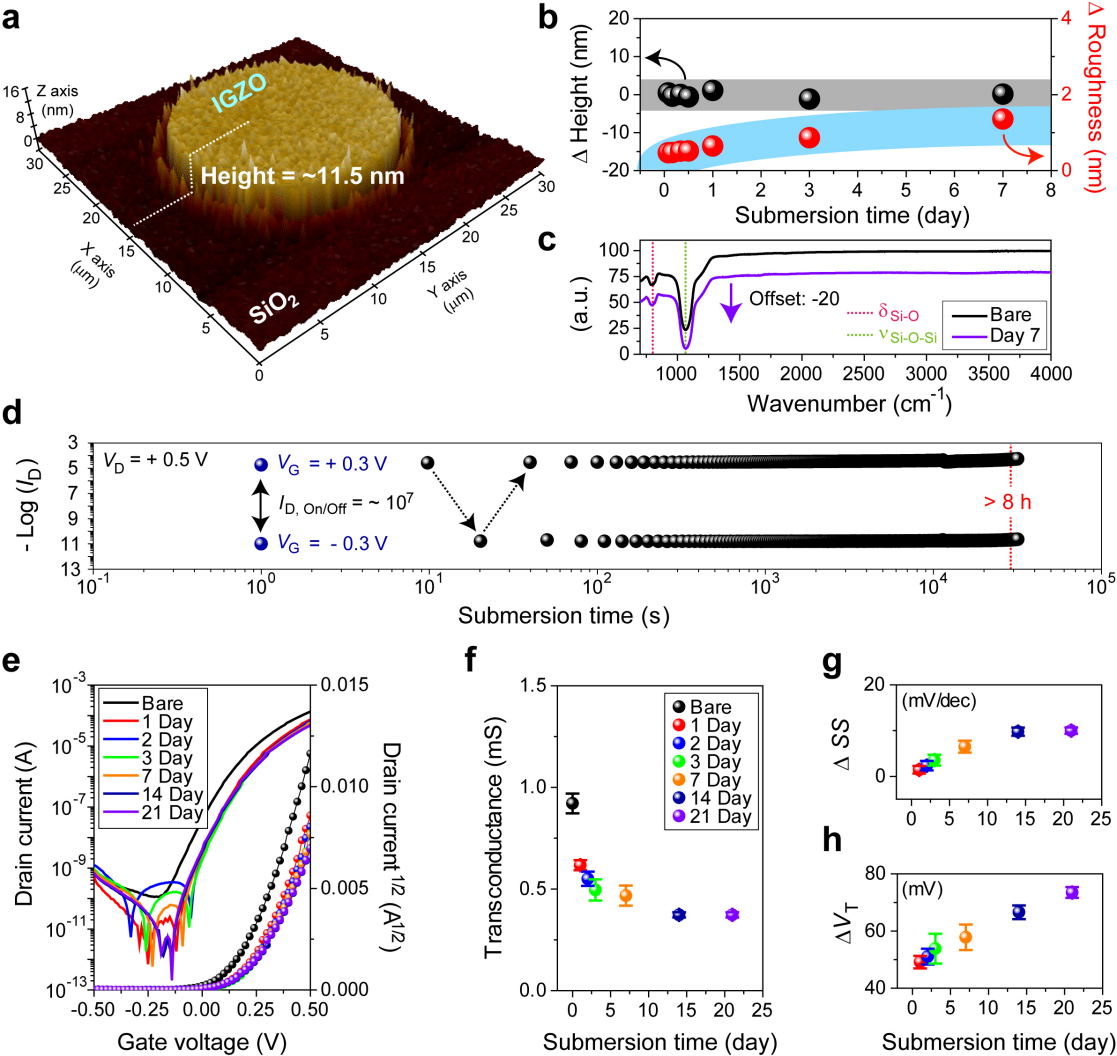
Material and electrical stability under physiological conditions. (a) Three-dimensional atomic force microscopy image of circularly patterned (*d* = 20 *μ*m) IGZO film on SiO_2_ substrates. (b) Height (black dots) and roughness (red dots) variations of IGZO films according to submersion time in DMEM under physiological conditions. (c) Attenuated total reflectance–Fourier transform infrared (ATR-FTIR) spectra of IGZO films before and after 7-day submersion. (d) Retention characteristics of submerged IGZO-EGTFTs, including measured source–drain currents (*I*_D_) at ±0.3 V gate bias (*V*_G_) over 8 h (Δ*t* = 10 s). (e) Representative transfer curves according to the submersion time (from 0 to 21 days). (f) Transconductance (*Δg*_m_), (g) subthreshold swing (*ΔSS*), and (h) threshold voltage (*ΔV*_T_) changes over submission time. The error bars indicate the standard deviations out of 20 individual devices.

The use of direct cellular interfaces necessitates the physicochemical durability of the active material and its long-term operational stability under physiological conditions, analogous to that in the human body. Therefore, the IGZO films with circular patterns (10 *μ*m radius) were prepared to measure the change in both surface roughness and thickness under physiological conditions [37 °C and 5% CO_2_; Fig. 2(a)]. Smooth (0.178 nm of root mean square roughness) and thin (11.5 nm thick) as-fabricated IGZO semiconducting films were submerged in DMEM and stored in a CO_2_ incubator where cells were grown following cultivation. After 1-week incubation, we observed negligible changes in surface height (variation of 0.14 nm; Fig. S2) and roughness (1.36 nm; Fig. S3) of the patterned IGZO films [Fig. 2(b)] and verified the presence of non-chemisorbed molecular species from DMEM electrolyte on the IGZO surfaces by attenuated total reflectance–Fourier transform infrared (ATR-FTIR) spectroscopy [Figs. 2(c) and S4]. These results indicate low chemical reactivity and long-term operational stability in an aqueous electrolyte solution with minimal metal oxide degradation.

We then analyzed the corresponding long-term electrical properties of IGZO-EGTFTs under physiological conditions to assess IGZO physicochemical resistivity. Before long-term electrical measurements, we confirmed that the operational characteristics were retained despite temperature changes ranging from room temperature (27 °C) to the typical incubator temperature (37 °C; Fig. S5). To examine the stability in device operation, positive- and negative-gate biases (*V*_G_ = +0.3 V [on], *V*_G_ = −0.3 V [off], *V*_D_ = +0.5 V) were periodically applied [Fig. 2(d)]. The resultant currents showed high switching ratios (>10^7^) without substantial deterioration over 8 h of extended retention and stable operation, even at an ultralow driving voltage (*V*_D_ = +0.01 V) associated with drain bias (Fig. S6).

It is also important to secure long-term IGZO-EGTFT electrical stability at the scale of days. The electrical characteristics of IGZO-EGTFTs stored under physiological conditions were preserved for over 3 weeks [Figs. 2(e)–2(h), with minimal changes in transconductance, subthreshold swing, and threshold voltage corresponding to variations Δ*g*_m_ = 0.5 mS, Δ*SS* = 10 mV/dec, and Δ*V*_T_ = 73 mV, respectively. These results demonstrate the excellent retention characteristics of EGTFTs under physiological conditions owing to the relatively higher stability of IGZO films than silicon or other metal oxide materials even in the presence of aqueous ionic solutions.^47,49,52–54^

### Cell compatibility of IGZO surfaces

**FIG. 3.**
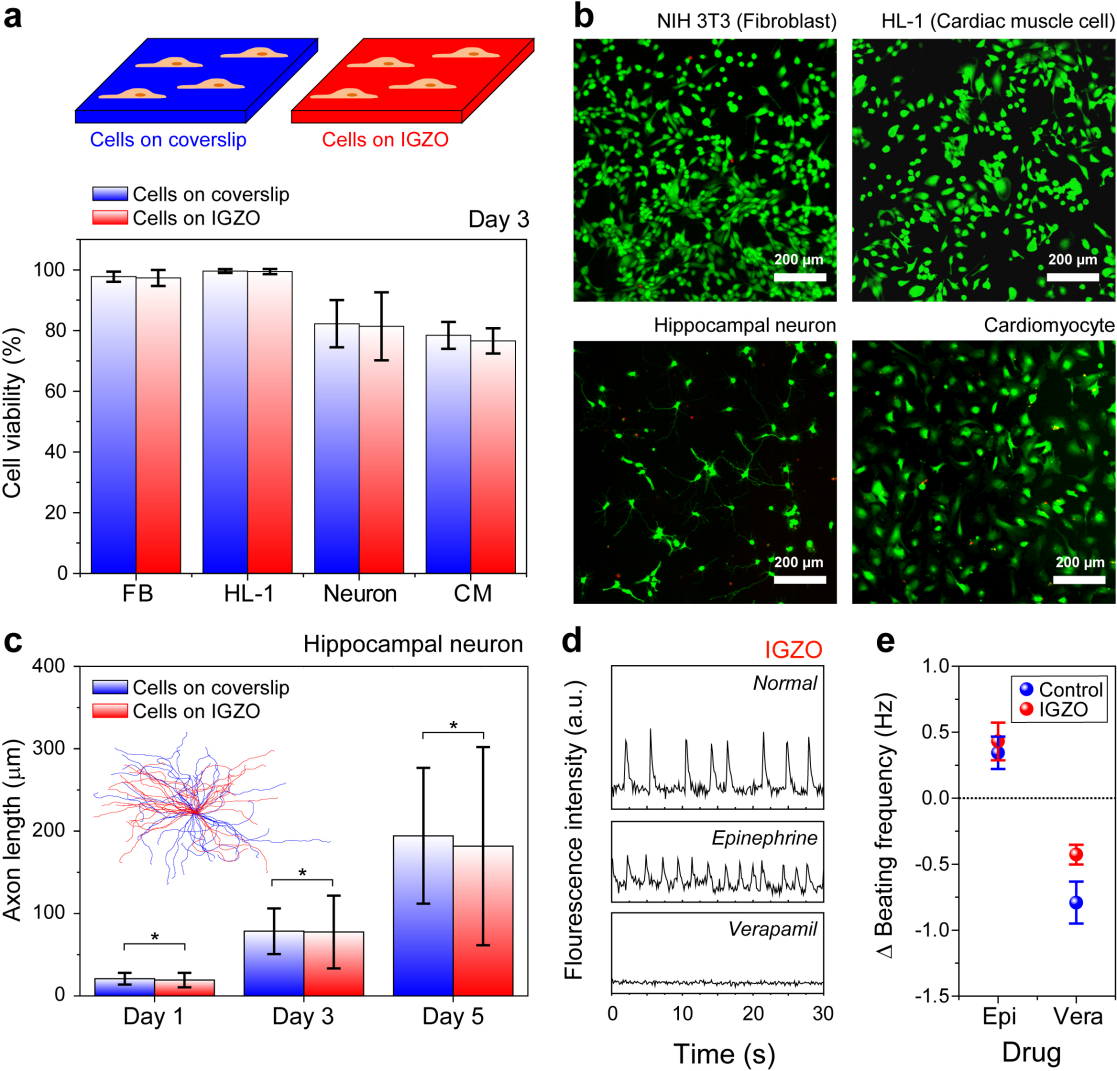
Analysis of cell viability and activities on IGZO surfaces. (a) Cell viability test on control (i.e., coverslips; blue) and IGZO samples (red) 3 days after cell seeding. Various mammalian cells (e.g., FB, NIH-3T3, fibroblasts; HL-1, cardiac muscle cells; neuron, embryonic rat hippocampal neurons; and CM, rat cardiomyocytes) were cultured. (b) Fluorescence image of mammalian cells stained with calcein acetoxymethyl (green) and ethidium homodimer-1 (red) on IGZO surface 3 days after cell seeding. (c) Plots of the average axonal length of hippocampal neurons cultured on coverslip (blue) and IGZO (red) at 1, 3, and 5 DIV (^*^p > 0.05, *n* = 50). Inset shows a collection of superimposed axon length traces of hippocampal neurons on control (blue) and IGZO samples (red) at 3 DIV. (d) Normalized spontaneous Ca^2+^ transients recorded from cardiomyocytes and acquired by time-lapse fluorescence imaging under different conditions. (e) Average variation in beating frequency (Epi and Vera denote cardiomyocyte treatment with epinephrine and verapamil, respectively). The error bars indicate the standard deviation across individual measurements from ten cardiomyocytes.

Before establishing an interface between live cells and semiconducting materials, the suitability of IGZO films for cell cultivation should be investigated in terms of cell viability and proliferation. Various mammalian cells, for example, NIH3T3, HL-1, rat hippocampal neurons, and rat cardiomyocytes, were cultured on IGZO films (see the Methods for details). Note that the NIH3T3 and HL-1 cells exhibited continuous cell divisions, so they were seeded to test cellular growth activities on IGZO surfaces. Further *in vivo* biocompatibility of IGZO films was evaluated by the primary cultured rat hippocampal neurons and cardiomyocytes, which could test neuronal outgrowth and monitor electroactivity on IGZO surfaces, respectively. The fluorescence microscopy was utilized for cell viability assessment. As shown in Figs. 3(a) and 3(b), NIH3T3 and HL-1 cells showed >95% viability, while primary neurons and cardiomyocytes showed >70% viability after 3 DIV (*days in vitro*) cultivation. Furthermore, fluorescent images after 3-day cultivation on IGZO surfaces revealed that various cell types exhibited growth and proliferation comparable to those observed in control experiments (Figs. S7–S10). Furthermore, the proliferations (counts/area) of NIH3T3 and HL-1 cells grown for 3 days on IGZO surfaces were similar to those grown on the control substrate (Figs. S7 and S8). Despite slightly lower NIH3T3 cell densities on IGZO than those on control after 5 days, Student's t-test showed that there are no significant statistical differences. Thus, we concluded that the IGZO surface supported the cultivation of various cell types with marginal toxicity.

In addition to cell viability and proliferation, we assessed morphological differences in the axons of hippocampal neurons and the beating rate of cardiomyocytes [Figs. 3(c)–3(e)]. Given that these cells exhibit electrophysiological activities and their dysfunction is closely related to neurological and physiological disorders, the potential negative effects associated with interfacing with metal oxide materials should be explored. At the developing stage, primary neuronal cells show the outgrowth of axons and dendrites from the cell body (i.e., soma). Figure 3(c) shows the quantitative analysis of axonal length and directionality obtained by immunostaining hippocampal neurons on control and IGZO at 1, 3, and 5 DIV (see also Fig. S11). We observed no significant differences in axonal outgrowth between neurons on control and IGZO samples. The randomly oriented axons growing without specific directionality indicated no specific chemical/physical cues provided by IGZO surfaces. Next, cardiomyocytes were cultured and their behaviors were analyzed in a similar way. The normal beating frequencies of cardiomyocytes can be promoted or inhibited by stimulation with drug additives. We applied epinephrine and verapamil as agonists to activate adrenergic receptors and block calcium channels, respectively, to see whether cardiomyocyte behaviors on IGZO surfaces are substantially different from those on the control. Based on calcium imaging results, the average beating frequencies of cardiomyocytes on the IGZO surfaces increased to 0.43 Hz after epinephrine treatment and decreased to −0.42 Hz after verapamil treatment [Figs. 3(d) and 3(e); see also Figs. S12, S13, and Movie S1]. Note that these metrics are comparable to those of the control (0.34 and −0.79 for epinephrine and verapamil, respectively). These results suggest that the IGZO layer supports the cultivation of various types of immortalized and primary cells without apparent cell toxicity or material-dependent peculiar behaviors and, potentially, is employed for sensing cellular activities directly on top (*vide infra*).

### Device characterizations of cell-interfaced IGZO-EGTFTs

**FIG. 4.**
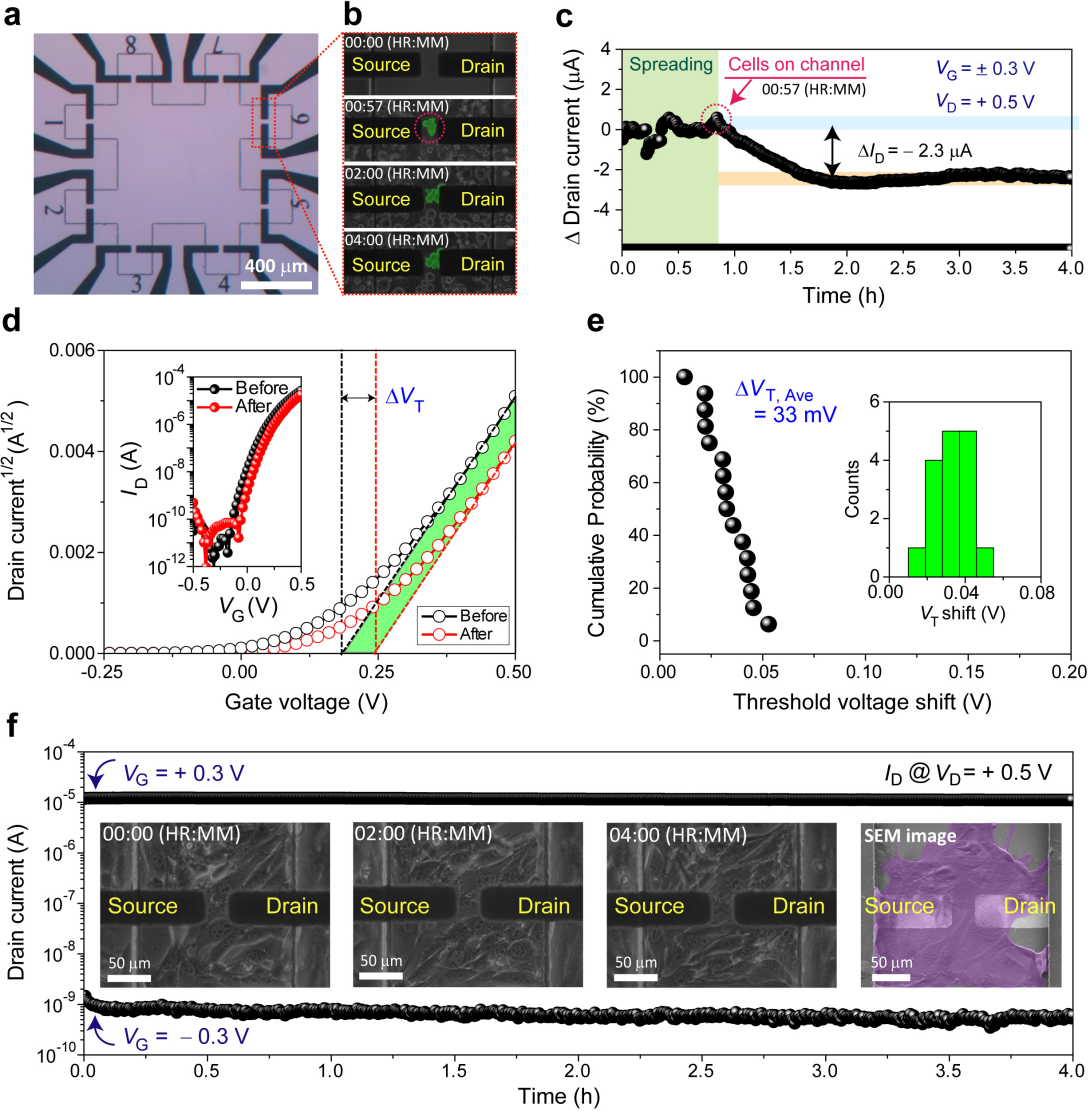
Direct cellular interfaces based on IGZO-EGTFTs. (a) Optical microscopy image of IGZO-EGTFTs. The channel length and width are 30 and 40 *μ*m, respectively. (b) Phase-contrast time-lapse images of fibroblasts from the initial cell seeding to the attachment. (c) Variation in the change of source–drain current (*ΔI*_D_) over time acquired every 10 s at +0.3 and −0.3 V of alternating gate biases (*V*_G_) with +0.5 V constant drain voltage (*V*_D_), corresponding to time-lapse imaging in (b). (d) Square root of drain current before cell seeding (black) and after 4 h of cell attachment (red) according to gate bias. The horizontal intercept indicates threshold voltages (*V*_T_) of each curve. *V*_T_ shift was observed after cell attachment. (e) Cumulative probability of *ΔV*_T_ after attachment of fibroblasts on IGZO channel regions. Inset: statistical distribution of 16 devices with the average of 33 mV. (f) Variation in *I*_D_ with the same manner in (c) with fibroblasts placed on top of IGZO-channel region. Inset: time-lapse images of mobile fibroblasts on IGZO surface with no indication of abnormality and representative scanning electron microscopy (SEM) image of fibroblasts on devices after cell compatibility measurements.

We fabricated an array of IGZO-EGTFTs with each channel length of 30 *μ*m and width of 40 *μ*m to monitor the behaviors of live fibroblast (NIH3T3) cells cultured on top. Note that NIH3T3 cells exhibited significant and continuous cell divisions; we evaluated the electrical performance as well as reliability of IGZO-EGTFTs in physiological relevant conditions with fibroblasts. The channel dimension was designed to make it comparable to the typical size of a mammalian cell so that each IGZO-EGTFT device could be sensitive to cellular-level activities [Figs. 4(a) and S14]. Subsequently, both the microscopic imaging of live cells and the real-time recording of transistor characteristics were conducted simultaneously [Figs. 4(b) and 4(c), see also Movie S2]. First, upon seeding fibroblasts (∼10^3^ cells/mm^2^) in the area defined by a circular glass well (I.D. ∼2.6 cm), time-lapse images were captured every 10 s, while the *I*_D_ was measured with the alternating gate bias scheme. We observed that the time-lapse Δ*I*_D_ started to decrease monotonously when the cells reached the IGZO channel after 57 min passed from the point of cell dispensing, and it was finally stabilized after the further decrease by ∼2.3 *μ*A when another 40 min passed out. Note that this result agrees with previous reports where the electrode under the two-dimensional extracellular matrices showed that the current settlement took similarly 40 min after cell attachment, which is mediated by the firm interaction with negatively charged cellular membrane.^15,20^

In parallel, we also monitored variations in *V*_T_ to correlate EGTFT characteristics and cell attachment. *V*_T_ is regarded as the minimum gate bias required for creating a conductive channel path between source and drain electrodes. Therefore, in this study, the measured *V*_T_ reflects environmental changes in the electrical field distribution near the interface between IGZO and aqueous electrolytes, which could be perturbed by fibroblast settlement on the channel. The IGZO-EGTFT transfer curves were recorded before/after fibroblast cell attachment for 4 h, which permits the full coverage over and the stabilized attachment of cells onto the IGZO channel of our interest. As shown in Fig. 4(d), the representative transfer curves as well as *V*_T_'s showed the positive shift. The statistical treatment of 16 different measurements confirmed the narrow distributions of positive *V*_T_ shift with an average of 33 mV [Fig. 4(e)]. This result can be attributed to the modulation of the surface charge density of IGZO by adherent cells with negatively charged plasma membranes, which is also implicated by a previous study.^15^

Finally, we examined the operational stability of IGZO-EGTFTs during the cell culture on top. For this purpose, we recorded the time-lapse source-drain current during the fibroblast attachment to the IGZO channel of our interest. Cells were incubated for 1 day after seeding to ensure the full coverage of the channel region. While normal fibroblast movements were imaged with a phase-contrast microscope, the *I*_D_ was measured with the alternating gate bias scheme [Fig. 4(f) and Movie S3]. The invariant electrical performance accompanying high degrees of cell compatibility demonstrated the excellent interface properties of the IGZO surface and its tolerance to cellular motion on top. Moreover, we demonstrated that IGZO-EGTFTs could be reused several times after the detachment of cells with trypsin (Figs. S15 and S16), which suggests that solution-processed IGZO-EGTFT is a cost-effective platform for direct cellular interfaces.

## CONCLUSIONS

We demonstrated the operation of large-area solution-processed IGZO-EGTFT arrays at low operating voltages (sub-0.5 V). The long-term material and operational stability of IGZO-EGTFT devices was successfully demonstrated owing to their robustness in the presence of aqueous electrolytes at physiological conditions. The biocompatibility of the IGZO surface to various types of mammalian cells was experimentally verified in terms of cell viability, proliferation, morphology, and drug responsiveness. Finally, the IGZO-EGTFT devices in direct contact with live cells showed prolonged stable operation as well as cell attachment dependent channel current and threshold voltages. We expect that these results provide the proof-of-concept of solution-processed metal oxide -based direct cellular interfaces, resulting in long-term stable implantable/on-chip devices, which can perform real-time monitoring of cellular behaviors, functions, and/or cell-derived biochemical.

## METHODS

### IGZO precursor solution synthesis

The IGZO precursors were prepared by dissolving indium nitrate hydrate [In(NO_3_)_3_·xH_2_O], gallium nitrate hydrate [Ga(NO_3_)_3_·xH_2_O], and zinc acetate dehydrate [Zn(CH_3_COO)_2_·2H_2_O] in 2-methoxyethanol. All reagents for synthesis were purchased from Sigma-Aldrich (St. Louis, MO, USA). After dissolving, the solution was vigorously stirred for more than 12 h at 75 °C and filtered through a 0.2 *μ*m polytetrafluoroethylene syringe filter (Sigma-Aldrich).

### IGZO-EGTFT fabrication

Quartz/Si wafers were cleaned using de-ionized water, acetone, and isopropanol, and then dried using nitrogen. Every substrate was a 2 cm^2^. The side-to-side (M-I-M) and top-gate bottom-contact configurations were selected for this study. Gold electrodes were fabricated by traditional photolithography and liftoff. The IGZO solution was spin-coated onto the electrode-deposited substrates at 3500 rpm for 30 s, and the coated substrate was thermally annealed on a hot plate at 350 °C for 1 h. To reduce parasitic and additional leakage currents, the IGZO thin films were patterned by photoresist photolithography and wet etching (LCE-12; Cyantek, USA). For electrical isolation, epoxy-based SU-8 (SU-8 2035, MicroChem) was used for passivation of the electrodes, except for the active semiconducting-layer regions. SU-8 photoresist was spin-coated at 5000 rpm for 40 s and prebaked at 65 °C for 2 min and then at 95 °C for 6 min to remove the solvent and anneal the SU-8 film, which was then exposed to ultraviolet light for 35 s under hard contact. We used an SU-8 developer (MicroChem) for 2 min as a photoresist developer. The thickness of the patterned SU-8 film was approximately 4 *μ*m.

### Electrical characterization by time-lapse imaging

The electrical characteristics were measured using a parameter analyzer (model 4200 SCS; Keithley, Cleveland, OH, USA) with pre-amplifier units under dark and physiological (37 °C with 5% CO_2_) conditions maintained by a cell-incubation system (Live Cell Instrument, Seoul, South Korea). Time-lapse images were captured using inverted fluorescence microscopy (IX 71; Olympus, Tokyo, Japan) under physiological conditions and processed by the ImageJ software (National Institutes of Health, Bethesda, MD, USA).

#### Cell culture

All cells were cultured on glass cover slips for control experiments and IGZO-coated silicon substrates. Each sample was placed in a 12-well plate. NIH3T3 and HL-1 cells were seeded as single cell lines. Neuronal cells were isolated from embryonic hippocampal tissue extracted from E18 Sprague Dawley rats, and cardiomyocytes were isolated from the heart tissue of 1 D postnatal rats by surgical dissection and dissociation. This research was approved by the Animal Care and Use committee of the Gwangju Institute of Science and Technology and followed their guideline (GIST-2022-047). For culturing HL-1 cells and embryonic hippocampal neurons, the surfaces of the substrates were coated with gelatin (HL-1) or poly-D-lysine (neurons). All the cell types were cultured with 2 × 10^4^ cells followed by incubation at 37 °C with 5% CO_2_.

#### Cell viability staining and imaging

The cells attached to the substrates were stained with 2 *μ*M calcein acetoxymethyl and 4 *μ*M ethidium homodimer-1 (Invitrogen, Carlsbad, CA, USA) for 15 min followed by washing three times with phosphate-buffered saline (PBS). Fluorescence microscopy (BX51WI; Olympus) was used to obtain fluorescence images of the stained live and dead cells, and image analysis was conducted using the ImageJ software (National Institutes of Health).

#### Immunofluorescence staining and imaging

The cells attached to the substrates were fixed with a 4% formaldehyde solution and 4% sucrose in PBS for 15 min at 25 °C (room temperature) and carefully washed three times with PBS. To permeabilize the cell membranes, samples were treated with 0.25% Triton X-100 for 10 min and washed three times with PBS. To prevent nonspecific biding of antibodies, all the samples were treated with 10% bovine serum albumin in PBS for 30 min at 37 °C and incubated for 90 min at room temperature with Tuj-1 primary antibody (1:200; sc-58888; Santa Cruz Biotech, Dallas, TX, USA) in 3% bovine serum albumin in PBS. The samples were then washed three times with PBS and incubated with Alexa-488-conjugated phalloidin (1:200; Invitrogen) and Alexa-546-conjugated secondary antibody (1:1000; Invitrogen) for 30 min at room temperature. Nuclear staining was conducted using 4′,6-diamidino-2-phenylindole (Invitrogen). Images were captured using a laser scanning confocal fluorescence microscope (FV1000; Olympus) and analyzed using the ImageJ software (National Institutes of Health).

#### Calcium imaging

Cardiomyocytes attached to the substrates were treated with 6 *μ*M Oregon Green 488 BAPTA-1 (Invitrogen) in PBS for 1 h for calcium imaging. To assess drug-induced function changes, 10 *μ*M verapamil and 10 *μ*M epinephrine (Sigma-Aldrich) were administered following treatment with 6 *μ*M Oregon Green 488 BAPTA-1 in each set of cardiomyocytes. All time-lapse fluorescence images were obtained by fluorescence microscopy (BX51WI; Olympus) at 8 frames/s and analyzed by the ImageJ software (National Institutes of Health).

#### Scanning electron microscopy

For cell fixation, substrates were treated with pre-warmed 4% glutaraldehyde solution for 15 min and washed three times with PBS. The samples were dehydrated with a graded series of ethanol (30%, 50%, 70%, 90%, and 100%) three times and dried using a critical-point dryer. The substrates were coated with platinum before imaging using a field-emission scanning electron microscope (6301F; JEOL, Tokyo, Japan).

## SUPPLEMENTARY MATERIAL

See the supplementary material for information that is not displayed in the main text, which includes the detailed analyses of structure/surface of solution-processed IGZO, electrical characterizations of resultant device under various condition, and live images/movies of cultured cells with simultaneously operating devices.

## Data Availability

The data that support the findings of this study are available from the corresponding authors upon reasonable request.
